# Integrating Radiogenomics and CSF-Based Liquid Biopsy Sequencing for Precision Neuro-Oncology

**DOI:** 10.3390/ijms27177619

**Published:** 2026-08-25

**Authors:** Klaudia Kubiak, Edyta Szurowska

**Affiliations:** 1 Second Department of Radiology, Medical University of Gdańsk, 80-210 Gdańsk, Poland; 2 Department of Radiology, University Clinical Centre, 80-210 Gdańsk, Poland

**Keywords:** radiogenomics, cerebrospinal fluid, liquid biopsy, next-generation sequencing, glioblastoma, glioma, circulating tumor DNA, precision neuro-oncology

## Abstract

Glioblastoma and diffuse gliomas pose major therapeutic challenges due to marked intratumoral heterogeneity, limited tissue accessibility, and the blood–brain barrier. Tissue-based next-generation sequencing (NGS) remains essential for WHO CNS5 molecular classification, yet it is invasive and poorly suited to serial monitoring. Two complementary non- or minimally invasive approaches have advanced rapidly: radiogenomics, which correlates multiparametric MRI features with genomic alterations, and cerebrospinal fluid (CSF) liquid biopsy sequencing, which detects circulating tumor DNA with high tissue concordance. This review examines the independent progress and synergistic integration of radiogenomics and CSF-NGS. Imaging signatures can non-invasively predict key drivers (*IDH1/2*, *EGFR*, *TERT*, *PTEN*, *TP53*) and molecular subtypes, while CSF-ctDNA sequencing enables real-time assessment of clonal evolution, therapy resistance (including post-temozolomide hypermutation), and residual disease. We discuss technical considerations, performance metrics, multimodal artificial-intelligence fusion, and emerging clinical applications for diagnosis, prognosis, treatment selection, and longitudinal surveillance. Critical challenges, standardization, prospective validation, and workflow integration are highlighted. By combining the spatial phenotypic information of radiogenomics with the temporal genomic resolution of CSF sequencing, this multimodal strategy offers a promising path toward precision neuro-oncology and reduced reliance on repeated invasive sampling.

## 1. Introduction

Glioblastoma (GBM), now defined as isocitrate dehydrogenase (IDH)-wildtype diffuse glioma of Central Nervous System (CNS) World Health Organization (WHO) grade 4, remains the most common and aggressive primary malignant brain tumor in adults [1,2]. Despite maximal safe resection followed by radiotherapy and temozolomide chemotherapy (the Stupp protocol), median overall survival is typically 12–18 months, and long-term survival is rare [1]. Adult diffuse gliomas as a group are characterized by marked inter- and intratumoral heterogeneity at the genetic, epigenetic, transcriptomic, and cellular levels [3,4]. This heterogeneity drives treatment resistance, clonal evolution, and inevitable recurrence, while the blood–brain barrier further limits drug delivery and complicates molecular monitoring [1,5].

Tissue-based next-generation sequencing (NGS) has become indispensable for integrated diagnosis according to the 2021 WHO Classification of Central Nervous System Tumors (WHO CNS5). It enables simultaneous detection of key alterations such as *IDH1/2* mutations, *TERT* promoter mutations, *EGFR* amplification, +7/−10 chromosome signature, *CDKN2A/B* homozygous deletion, and gene fusions [2,6,7]. However, tissue sampling is inherently limited. Surgical resection or stereotactic biopsy is invasive, carries neurological risk, and is not always feasible, particularly in elderly or frail patients or those with deep-seated lesions [8]. Even when tissue is obtained, a single biopsy frequently fails to capture the full spatial heterogeneity of the tumor, leading to sampling bias and incomplete molecular profiles [3,9]. Moreover, repeated tissue sampling for longitudinal monitoring of clonal evolution or therapy resistance is rarely practical [10].

These constraints have accelerated the development of two complementary non- or minimally invasive approaches. Radiogenomics links quantitative multiparametric magnetic resonance imaging (MRI) features (radiomics) with underlying genomic and molecular alterations, offering the prospect of a “virtual biopsy” that can predict *IDH* status, *EGFR* alterations, molecular subtypes, and other clinically relevant biomarkers without tissue sampling [11,12]. In parallel, cerebrospinal fluid (CSF) liquid biopsy sequencing has emerged as a superior source of circulating tumor DNA (ctDNA) compared with plasma, owing to direct contact with the tumor and higher concentrations of tumor-derived nucleic acids. CSF- NGS achieves detection rates of approximately 50–80% and high concordance with tissue genotypes for key driver mutations, enabling molecular characterization, monitoring of residual disease, and early detection of resistance mechanisms [13,14,15,16].

The integration of radiogenomics and CSF-based liquid biopsy sequencing is particularly compelling. Radiogenomics provides spatially resolved phenotypic information that can guide interpretation of liquid biopsy findings and potentially predict which patients are most likely to shed detectable ctDNA. Conversely, CSF sequencing supplies temporal genomic resolution that imaging alone cannot achieve. Together, these modalities can reduce reliance on repeated invasive procedures, improve molecular classification when tissue is limited, support real-time monitoring of treatment response and resistance, and facilitate adaptive therapeutic strategies [13,14,15,16,17].

**Figure 1:**
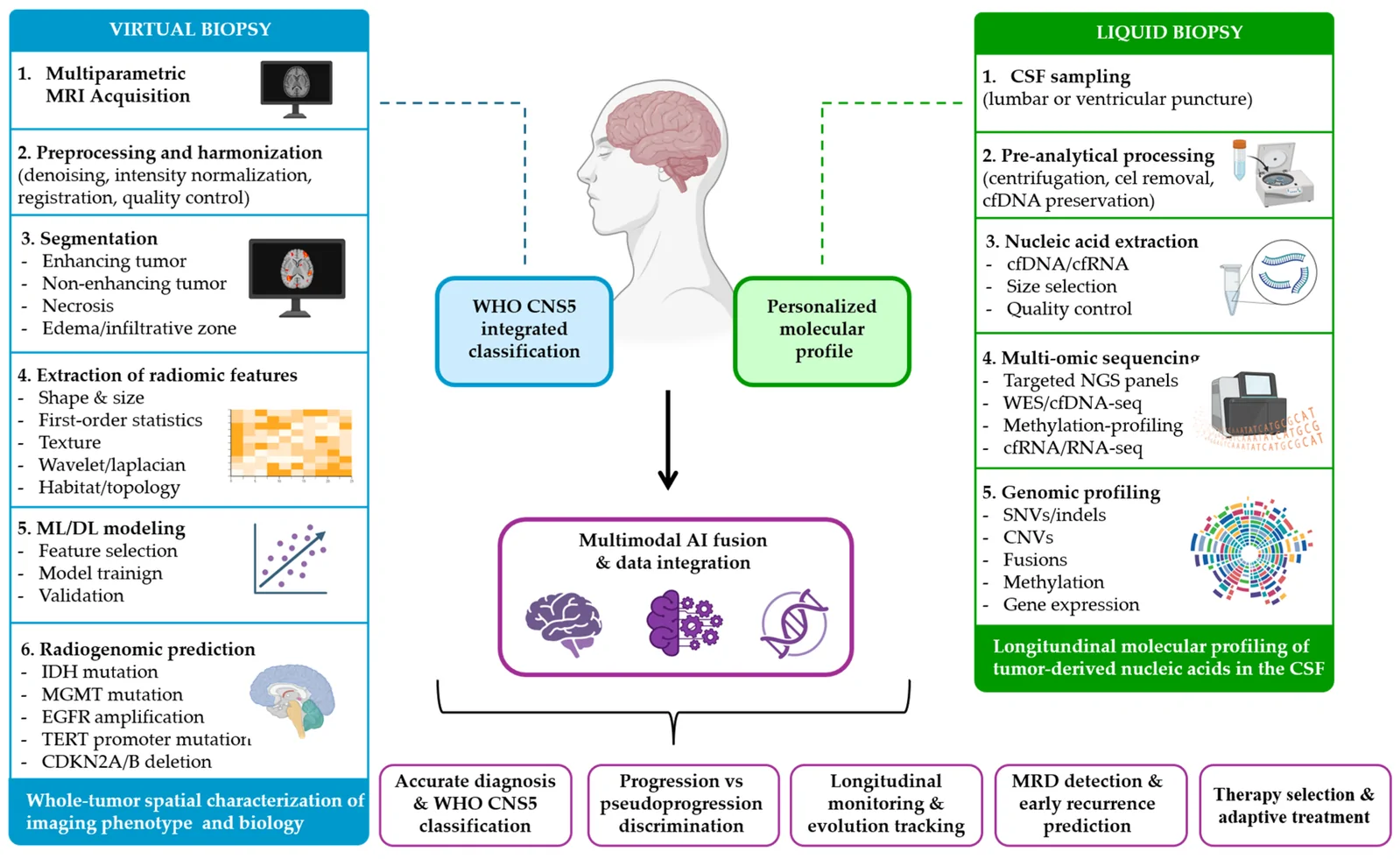
Integrated Virtual and Liquid Biopsy Framework for Precision Neuro-Oncology. Schematic overview of the proposed multimodal framework integrating radiogenomics and cerebrospinal fluid (CSF)-based liquid biopsy for the comprehensive characterization and longitudinal monitoring of adult diffuse gliomas. Multiparametric MRI-derived radiogenomic signatures provide spatially resolved phenotypic information across the tumor, whereas CSF-based next-generation sequencing (CSF-NGS) provides longitudinal genomic and molecular information. These complementary data streams can be integrated using artificial intelligence and multimodal data-fusion approaches to support molecular classification, treatment selection, assessment of treatment response, minimal residual disease detection, and precision clinical decision-making. Created in BioRender. Kubiak, K. (2026) https://BioRender.com/nb3pbt9, accessed on 5 August 2026.

Although previous reviews have independently examined radiogenomics or CSF-based liquid biopsy in glioma, the present review specifically focuses on their integration into a complementary ‘virtual + liquid biopsy’ framework (Figure 1). We emphasize how spatially resolved imaging phenotypes and longitudinal molecular information from CSF can be interpreted together to address clinical questions that may remain unresolved by either modality alone. Particular attention is given to patient-level applications, including molecular characterization when tissue is unavailable, treatment stratification, assessment of suspected progression, and early detection of molecular relapse.

## 2. Foundations of Radiogenomics in Brain Tumors

Radiomics refers to the high-throughput extraction of quantitative features from medical images that capture tumor morphology, intensity, texture, and higher-order patterns beyond visual assessment [18,19]. Radiogenomics extends this concept by correlating these imaging phenotypes with underlying genomic, transcriptomic, or epigenomic alterations, thereby enabling non-invasive inference of molecular characteristics [12,20]. The central premise is that imaging phenotypes reflect the cumulative effects of genetic and molecular processes, including angiogenesis, cellular density, invasion, and necrosis [11].

Standard radiogenomic workflows begin with multiparametric MRI acquisition. Conventional sequences typically include T1-weighted (pre- and post-contrast), T2-weighted, fluid-attenuated inversion recovery (FLAIR), and diffusion-weighted imaging. Advanced sequences such as perfusion-weighted imaging (dynamic susceptibility contrast or dynamic contrast-enhanced), diffusion tensor imaging, and, less commonly, MR spectroscopy or chemical exchange saturation transfer further enrich the phenotypic information [12,21]. After preprocessing (resampling, intensity normalization, skull-stripping), regions of interest—commonly the contrast-enhancing tumor, non-enhancing FLAIR hyperintensity, and necrotic core—are segmented manually or via automated deep-learning tools. From these volumes, hundreds to thousands of radiomic features (shape, first-order statistics, gray-level co-occurrence matrices, run-length matrices, and wavelet or Laplacian-of-Gaussian filtered features) are extracted [19,22].

Machine-learning and deep-learning models are then trained to map these features to NGS-defined alterations. Classical approaches (random forests, support vector machines, elastic-net logistic regression) remain widely used, while convolutional neural networks and hybrid radiomics–deep-learning architectures increasingly dominate newer studies [12,23]. Performance varies by biomarker. *IDH* mutation status is consistently the most accurately predicted alteration, with area-under-the-curve (AUC) values frequently exceeding 0.90 in single-institution cohorts and remaining robust (AUC ≈ 0.75–0.90) in multi-center external validation [12,24,25]. *EGFR* amplification or mutation prediction is more modest (AUC typically 0.70–0.85), reflecting greater spatial heterogeneity of EGFR-driven phenotypes [12,26]. *TERT* promoter mutation models achieve AUCs of approximately 0.80–0.90 when multiparametric data are combined with clinical variables [27]. *MGMT* promoter methylation prediction remains challenging, with reported AUCs often in the 0.65–0.80 range and limited generalizability [25,28]. Prediction of *CDKN2A/B* homozygous deletion and broader molecular subtypes (classical, mesenchymal, proneural) is feasible but generally less accurate than *IDH* classification [11,23].

Spatiotemporal considerations are critical. Recent work has shown that radiogenomic signatures are stronger in molecularly homogeneous tumors and become diluted when multiple co-occurring drivers are present [11]. Tumor location itself carries genomic information (spatiogenomics): certain driver alterations exhibit preferential anatomic distributions, and integrating location-derived features can improve model performance [11,25]. Longitudinal imaging further enables tracking of phenotypic evolution that parallels clonal changes detected by sequential NGS, although formal radiogenomic–temporal studies remain limited.

Despite these advances, important limitations persist. Most published models are trained on relatively small, single-institution cohorts, frequently underpowered relative to feature dimensionality, which predisposes to overfitting [16,29]. External validation is still uncommon, and performance often declines when models are applied across scanners, field strengths, or acquisition protocols [25,27,29]. Interpretability remains incomplete: while some radiomic features can be linked to biological processes (e.g., high perfusion to angiogenesis in *EGFR*-amplified tumors), many high-performing signatures lack clear mechanistic grounding [21]. Standardization of segmentation, feature extraction (e.g., adherence to Image Biomarker Standardization Initiative guidelines), and reporting is improving but not yet universal [18,22].

In summary, radiogenomics has matured into a powerful framework for non-invasive prediction of key NGS-defined biomarkers in gliomas, with particularly strong performance for *IDH* status. Continued progress will depend on larger multi-institutional datasets, rigorous external validation, improved biological interpretability, and tighter integration with liquid-biopsy NGS data. A comparative overview of the most extensively investigated NGS-defined biomarkers, including representative diagnostic performance, imaging features, and current limitations, is provided in Table 1.

## 3. CSF-Based Liquid Biopsy Sequencing: Technical and Biological Basis

ctDNA released by glioma cells into biofluids offers a minimally invasive window into tumor genomics. In CNS tumors, cerebrospinal fluid (CSF) consistently outperforms plasma as a source of tumor-derived nucleic acids. The blood–brain barrier restricts the passage of tumor DNA into the systemic circulation, resulting in very low plasma ctDNA fractions (typically <1%). In contrast, CSF is in direct contact with the tumor microenvironment and contains substantially higher concentrations of tumor-derived DNA (tumor fractions often reaching 10–50%) [13,14,44]. Meta-analyses confirm this advantage: pooled CSF-ctDNA detection rates reach approximately 70–82%, compared with only 14–16% in matched plasma samples, with molecular concordance to tissue genotypes of 84–90% [14,45].

Pre-analytical variables critically influence assay performance. CSF may be obtained via lumbar puncture, intraoperative sampling (cisternal, ventricular, or resection-cavity), or reservoir (Ommaya) aspiration. While detection rates are broadly comparable across routes, proximity to the tumor can enrich tumor DNA yield [46,47]. Immediate processing is essential because cell-free DNA has a short half-life. Standard protocols involve centrifugation to remove cellular debris, followed by extraction using silica-column or magnetic-bead methods; bead-based approaches generally recover a higher proportion of short fragments characteristic of tumor DNA [46,47]. Storage at −80 °C preserves analyte integrity for downstream sequencing. Recent consensus recommendations emphasize standardized reporting of collection site, processing time, centrifugation parameters, and extraction kit to improve reproducibility across studies [46].

NGS platforms for CSF analysis range from focused targeted panels to more comprehensive approaches. Many clinical and research assays employ custom or commercial panels covering recurrent glioma drivers (*IDH1/2*, *TERT* promoter, *EGFR*, *PTEN*, *TP53*, *ATRX*, *BRAF*, *H3K27M*, and others), often with unique molecular identifiers and ultra-deep sequencing (coverage > 5000–10,000×) to detect low-variant-allele-frequency (VAF) mutations [13,45,48]. Hybrid-capture or amplicon-based methods predominate. Emerging strategies incorporate methylation profiling, fragmentomics, and simultaneous DNA/RNA sequencing to expand molecular coverage [49]. Multi-omic CSF assays that combine mutation detection with copy-number variation and epigenetic signatures are under active development and may improve sensitivity in low-shedding tumors.

In practice, the most frequently detected alterations in CSF-ctDNA mirror the genomic landscape of diffuse gliomas: *EGFR* (amplification or mutation), *PTEN*, *TP53*, *IDH1*, *TERT* promoter, and *PIK3CA* are among the most common findings [13,45]. Detection rates are generally higher in high-grade and *IDH*-wildtype tumors, although *IDH*-mutant gliomas also yield informative results. Tissue–CSF concordance for shared mutations is high (typically > 80%), supporting the use of CSF as a surrogate when tissue is unavailable or limited [13,14].

Clinically, CSF sequencing is valuable both at diagnosis and during disease course. In newly diagnosed patients, especially those for whom biopsy is high-risk or infeasible, CSF-NGS can provide molecular classification aligned with WHO CNS5 criteria and identify potentially actionable alterations [34]. At recurrence, serial CSF sampling enables detection of clonal evolution and therapy-induced resistance mechanisms, including mismatch-repair (MMR) gene mutations (*MSH2*, *MSH6*, *MLH1*, *PMS2*) that arise after alkylating-agent exposure and drive hypermutation [17]. CSF-ctDNA quantification (VAF or tumor fraction) is being explored as a marker of minimal residual disease (MRD) and as a tool to distinguish true progression from pseudoprogression, although prospective validation remains ongoing [13,45].

Beyond ctDNA, additional CSF analytes are expanding the liquid-biopsy toolkit. Circulating tumor RNA (ctRNA), including fusion transcripts, can be co-extracted and sequenced, improving detection of rearrangements [49]. Extracellular vesicles (exosomes) carry tumor-derived DNA, RNA, and proteins and may cross the blood–brain barrier more readily than free nucleic acids. Methylation signatures of cell-free DNA offer independent diagnostic and prognostic information and can complement mutation-based assays [16,50]. Multi-analyte approaches that integrate these signals are expected to increase overall sensitivity and biological insight.

CSF-based testing has several clinically important limitations. Tumor-derived DNA may be undetectable in patients with low-grade disease, low-shedding molecular subtypes, small tumor volume, or lesions that are remote from the ventricular system and major subarachnoid CSF spaces [13,14,45]. Sampling site therefore matters: lumbar CSF may not accurately represent a lesion with limited communication with the sampled compartment, whereas ventricular, cisternal, or intraoperative samples may have higher tumor fractions but are less readily obtained [13,14,44,46,47]. Timing is also relevant, because CSF collected before surgery, after resection, during radiotherapy, or at recurrence may contain different amounts and compositions of tumor-derived DNA [13,16,45,48]. A negative result should consequently be reported as “no alteration detected above the assay’s limit of detection,” rather than as molecular negativity. Lumbar puncture may be unsuitable in patients with suspected obstructive hydrocephalus, substantial intracranial mass effect, impaired CSF circulation, coagulopathy, infection at the puncture site, or other individualized procedural risks [8,44]. Clinical decisions should therefore incorporate neuroimaging, neurological examination, platelet and coagulation status, and neurosurgical or neuroradiological assessment.

In summary, CSF-based liquid biopsy sequencing provides a high-fidelity, minimally invasive means of interrogating glioma genomics. Its superiority over plasma, improving technical standardization, expanding assay designs, and growing clinical applications position it as a cornerstone technology for precision neuro-oncology, particularly when combined with complementary modalities such as radiogenomics. Collectively, prospective studies and recent meta-analyses consistently demonstrate higher ctDNA detection rates and greater tissue concordance in CSF compared with plasma. A summary of the principal clinical studies and their reported molecular findings is presented in Table 2.

## 4. Synergistic Integration of Radiogenomics and CSF NGS

Radiogenomics and CSF-based liquid biopsy sequencing provide complementary layers of information that, when combined, address key limitations of each modality alone. Radiogenomics delivers spatially resolved phenotypic data across the entire tumor volume, capturing regional differences in cellularity, angiogenesis, necrosis, and invasion that reflect underlying molecular heterogeneity [11,19]. CSF-NGS, by contrast, supplies high-resolution genomic and, increasingly, multi-omic snapshots of tumor-derived material that has entered the cerebrospinal fluid compartment, enabling detection of specific mutations, copy-number changes, and evolutionary trajectories over time [13,14,44]. Conceptually, imaging functions as a whole-tumor “spatial map,” while CSF sequencing functions as a dynamic “genomic sensor.” Their integration therefore offers both anatomic context and temporal molecular resolution—attributes that neither approach can fully achieve independently [16,54]. Radiogenomics and CSF sequencing should not be viewed as competing technologies. Instead, they provide fundamentally different yet complementary information regarding tumor biology. Whereas radiogenomics offers non-invasive whole-tumor spatial characterization, CSF- NGS enables longitudinal molecular monitoring. Their respective strengths and limitations are summarized in Table 3.

Radiogenomic signatures may help guide the timing, site, or interpretation of CSF sampling. Tumors with imaging features associated with high cellular turnover, disruption of the blood–brain barrier, or proximity to CSF spaces (ventricles or subarachnoid cisterns) are more likely to shed detectable ctDNA [11,55]. Conversely, radiogenomic models that predict low tumor fraction or molecularly quiescent phenotypes could flag cases in which a negative CSF result is more likely to represent true absence of detectable ctDNA rather than technical failure. In the longitudinal setting, changes in radiomic features (e.g., increasing contrast enhancement or diffusion restriction) may prompt earlier CSF sampling to investigate clonal evolution or resistance before clinical deterioration becomes evident.

Multimodal artificial-intelligence frameworks are beginning to operationalize this complementarity. Early studies have demonstrated that combining radiomic features with liquid-biopsy analytes (including plasma or CSF-derived miRNA, methylation, or proteomic profiles) improves outcome prediction compared with either modality alone [56,57]. Although dedicated radiogenomic–CSF-ctDNA fusion models remain limited, the same deep-learning and multi-view learning architectures successfully applied to radiomics-plus-clinical or radiomics-plus-genomic tissue data can be extended to incorporate CSF-NGS variant allele frequencies, tumor fractions, and mutation spectra. Such models could generate integrated probability scores for molecular subtype, residual disease burden, or risk of early progression.

Several high-value clinical scenarios illustrate the potential of this integration:

1. Non-invasive molecular classification when tissue is limited or high-risk. In elderly patients, deep-seated lesions, or cases where biopsy carries unacceptable morbidity, a radiogenomic prediction of *IDH1/2* or *EGFR* status can be corroborated or refined by CSF-NGS, increasing diagnostic confidence and supporting WHO CNS5-aligned classification without tissue [16,47].
2. Differentiation of true progression from pseudoprogression. Conventional MRI frequently cannot distinguish these entities. Radiomic signatures of progressive disease combined with rising CSF-ctDNA VAF or the appearance of new resistance mutations would favor true progression, whereas stable or declining CSF signals would support treatment-related changes [13,45].
3. Tracking clonal evolution and enabling adaptive therapy. Serial multiparametric MRI and CSF sampling can monitor the emergence of therapy-induced alterations (e.g., MMR-gene mutations after temozolomide) and spatially map regions of phenotypic change that may correspond to resistant clones, thereby informing decisions about treatment intensification, switch, or trial enrollment [44,49].
4. Prognostication and clinical-trial stratification. Integrated radiogenomic–CSF scores that incorporate imaging-derived heterogeneity metrics and CSF tumor fraction or mutation burden may improve risk stratification beyond either modality alone and help select patients for targeted or immunotherapy trials [10,58].

At present, fully integrated prospective studies remain scarce. Most evidence is still parallel rather than fused: radiogenomic models and CSF-NGS assays have each been validated independently against tissue NGS, and narrative reviews have highlighted their complementary strengths [16,55]. Proof-of-concept multimodal work has largely involved radiomics combined with plasma or CSF multi-omics (methylation, proteomics, miRNA) rather than mutation-level CSF-NGS [56,58]. Nevertheless, the technical foundations are in place, guideline support for CSF testing in biopsy-infeasible cases is expanding [47], and computational methods for multi-modal data fusion are rapidly maturing. Coordinated multi-institutional efforts that prospectively collect paired multiparametric MRI, CSF, and tissue will be essential to move from conceptual synergy to clinically validated integrated biomarkers.

The complementary roles of radiogenomics and CSF sequencing are expected to provide the greatest clinical benefit in selected high-impact scenarios. The principal applications, expected clinical value, and current level of evidence are summarized in Table 4.

## 5. Clinical Utility and Translational Potential

The integration of radiogenomics and CSF-based liquid biopsy sequencing holds substantial promise for improving diagnosis, monitoring, and treatment selection in adult diffuse gliomas, particularly glioblastoma. Tissue-based NGS remains the reference standard for WHO CNS5 integrated diagnosis; however, when tissue is limited, non-diagnostic, or unobtainable, combined radiogenomic prediction and CSF-NGS can supply complementary molecular information that supports classification and clinical decision-making [2,16,48]. Radiogenomic models that accurately predict *IDH1/2* status, *EGFR* alterations, or molecular subtype can be corroborated by CSF-ctDNA detection of the corresponding mutations, increasing diagnostic confidence in biopsy-infeasible cases. Recent updates to NCCN Clinical Practice Guidelines now explicitly recommend CSF-based molecular tumor profiling for high-grade glioma and glioblastoma when tissue biopsy is not feasible, reflecting growing acceptance of this approach in routine care pathways [59].

In clinical practice, the integrated approach would be applied selectively rather than as a universal replacement for tissue diagnosis. At initial presentation, a patient would undergo standardized multiparametric MRI and multidisciplinary assessment of surgical and biopsy feasibility. When resection or stereotactic biopsy is unsafe, nondiagnostic, or unlikely to provide adequate tissue, CSF sampling could be considered if there is no contraindication to lumbar puncture and if the lesion is anatomically likely to shed tumor-derived DNA into the CSF [8,13,16,44]. CSF analysis should preferably be obtained before treatment, or at a clearly documented clinical landmark, because surgery, radiotherapy, corticosteroids, and systemic therapy may alter tumor shedding and the detectable molecular profile [13,14,45,48].

The MRI-derived radiogenomic output would provide a spatial phenotype and a probabilistic estimate of selected molecular features, whereas CSF-NGS would provide direct evidence of detectable alterations, clonal composition, and tumor fraction [11,13,14,16,44,45]. Results should be reviewed in a molecular tumor board together with clinical findings, conventional pathology, tissue sequencing when available, and the limitations of each assay. A negative CSF result should not be interpreted as evidence that the tumor lacks a particular alteration, especially in tumors with low shedding, remote ventricular or subarachnoid contact, or low tumor fraction [13,14,45]. Similarly, a radiogenomic prediction should be regarded as supportive rather than definitive until externally validated for the relevant scanner, population, and clinical setting [25,27,28,29,56].

During follow-up, CSF sampling would be considered when MRI findings are equivocal, when there is suspected progression, when treatment resistance is clinically suspected, or when molecular relapse could alter management [13,16,45,48]. A concordant increase in radiomic risk features and CSF tumor fraction or variant allele frequency would support multidisciplinary consideration of treatment change or clinical-trial enrollment [13,45,58]. Discordant results would generally prompt repeat imaging, repeat CSF analysis when clinically appropriate, or tissue sampling if safe, rather than an immediate treatment decision based on one modality alone [8,13,16,47].

During and after standard therapy (maximal safe resection followed by radiotherapy with concurrent and adjuvant temozolomide), serial multiparametric MRI and CSF sampling offer a practical strategy for disease monitoring. Conventional imaging alone struggles to distinguish true progression from pseudoprogression or radiation necrosis. Rising CSF-ctDNA variant allele frequency or the appearance of new mutations, when interpreted alongside radiomic changes suggestive of progressive disease, can increase specificity for true progression and guide earlier therapeutic adjustments [13,45]. Conversely, declining or undetectable CSF-ctDNA in the setting of stable or improving imaging features may support continued observation or de-escalation. Although prospective data remain limited, early evidence indicates that higher CSF-ctDNA burden is associated with shorter progression-free and overall survival, supporting its potential role as a dynamic prognostic biomarker [13].

Both modalities can facilitate access to targeted therapies and clinical trials. Identification of actionable alterations (*BRAF V600E*, *NTRK* fusions, high tumor mutational burden, or emerging targets) via CSF-NGS, especially when tissue is unavailable, can enable enrollment in molecularly matched studies or off-label use of approved agents under appropriate circumstances [6,48]. Radiogenomic signatures that stratify patients by predicted molecular subtype or heterogeneity may further refine trial eligibility and help enrich cohorts for therapies directed at specific biological phenotypes. Integration of the two approaches could therefore improve the efficiency of precision-oncology pipelines in neuro-oncology, where actionable alterations are relatively infrequent and tissue access is often constrained.

Cost-effectiveness and workflow considerations are still evolving. CSF collection via lumbar puncture is less resource-intensive than surgical biopsy but is not risk-free and requires appropriate clinical infrastructure. Advanced multiparametric MRI and radiomic analysis demand standardized acquisition protocols, computational resources, and expertise that are not universally available. Formal health-economic evaluations specific to combined radiogenomic–CSF strategies in glioma are lacking; however, the potential to avoid unnecessary surgeries, reduce ineffective treatment courses, and accelerate appropriate trial enrollment may offset incremental testing costs in selected populations. Streamlined workflows that embed CSF sampling at the time of diagnostic lumbar puncture or intraoperative procedures, coupled with automated radiomic pipelines, will be important for scalability.

From a regulatory and implementation perspective, the landscape is advancing. The recent expansion of NCCN recommendations to include CSF genomic profiling in biopsy-infeasible high-grade gliomas and glioblastomas marks a significant milestone [59]. Commercial CSF-based comprehensive genomic profiling assays have undergone analytical and clinical validation and are increasingly accessible. Radiogenomic tools, while still largely research-oriented, are progressing toward clinical-grade software with improved standardization (e.g., adherence to Image Biomarker Standardization Initiative guidelines). Key remaining barriers include prospective multi-center validation of integrated models, reimbursement pathways, inter-laboratory harmonization of CSF-NGS assays, and education of multidisciplinary teams. Coordinated efforts among neuro-oncologists, neurosurgeons, neuroradiologists, molecular pathologists, and regulatory bodies will be required to translate the complementary strengths of radiogenomics and CSF sequencing into routine precision neuro-oncology practice.

### 5.1. Patient-Level Clinical Decision Scenarios

To illustrate how the integrated “virtual + liquid biopsy” framework could influence individual patient management, we outline four representative clinical scenarios. These examples emphasize the complementary nature of radiogenomics and CSF-NGS and highlight the need for multidisciplinary interpretation.

#### 5.1.1. Biopsy-Infeasible or Insufficient Tissue

In patients in whom surgical biopsy is considered unsafe, technically infeasible, or unlikely to provide adequate diagnostic material, the combination of radiogenomic imaging and CSF-based molecular profiling may provide complementary information [6,13,14,15,48,59]. Radiogenomic analysis can characterize the spatial phenotype and estimate the probability of relevant molecular alterations, while CSF-NGS may provide direct evidence of tumor-derived genomic alterations when tissue is unavailable or insufficient [6,13,14,15,17,48,59]. Concordant findings could increase confidence in molecular characterization and support treatment or clinical-trial decisions when tissue is unavailable. Importantly, such an approach should complement rather than replace tissue-based diagnosis whenever tissue sampling is clinically feasible and appropriate.

#### 5.1.2. Discordant MRI and CSF Findings

Discordance between imaging and CSF findings represents an important clinical scenario in which integrated interpretation may be particularly valuable [13,14,15,16,17]. For example, radiomic features may suggest an aggressive or progressive phenotype while CSF-ctDNA remains stable or undetectable. Because CSF-ctDNA shedding is variable and negative CSF-ctDNA does not exclude the presence of tumor, such discordance should not automatically lead to treatment de-escalation; instead, repeat imaging and/or CSF analysis and multidisciplinary reassessment may be appropriate [13,14,15,16,17]. Conversely, increasing CSF-ctDNA in the absence of convincing radiological progression may indicate emerging molecular activity and justify intensified surveillance [13,14,15,16,17].

#### 5.1.3. Suspected Pseudoprogression

The distinction between treatment-related imaging changes and true tumor progression is another potential application of integrated radiogenomic and CSF-based assessment [13,14,15,16,17]. In a patient with new or increasing contrast enhancement after chemoradiotherapy, radiomic and multiparametric MRI features may provide additional information regarding the likelihood of progressive tumor [50]. Concurrently, increasing CSF-ctDNA levels or the emergence of resistance-associated alterations may provide molecular evidence supporting active disease [13,14,15,16,17]. Conversely, stable or declining CSF-ctDNA in the setting of equivocal imaging could support continued observation and short-interval reassessment rather than an immediate change in therapy [13,14,15,16,17]. These interpretations should remain contextual and should not be considered definitive in the absence of prospective validation.

#### 5.1.4. Molecular Relapse Before Radiological Progression

A further potential application is surveillance for molecular relapse. A patient may show stable conventional MRI findings while serial CSF analysis demonstrates newly detectable or increasing tumor-derived DNA or emerging molecular alterations [13,14,15,16,17]. In such a setting, the integrated result could prompt closer radiological and molecular surveillance, multidisciplinary review, and consideration of clinical-trial enrollment or other early interventions when supported by the molecular findings [13,14,15,16,17,48]. This potential for molecular detection before overt radiological progression represents one of the major conceptual advantages of longitudinal CSF analysis, but its clinical benefit remains to be prospectively established [13,14,15,16,17].

## 6. Challenges, Limitations, and Standardization Needs

Despite encouraging technical and clinical progress, several interconnected challenges must be addressed before the integrated use of radiogenomics and CSF-based liquid biopsy sequencing can be widely adopted in precision neuro-oncology.

Technical challenges. CSF-ctDNA assays still face sensitivity limitations in tumors with low shedding rates; detection is not universal even in high-grade gliomas, and negative results cannot reliably exclude disease [13,14,45]. Inter-platform variability in library preparation, sequencing depth, bioinformatic pipelines, and variant-calling thresholds complicates cross-study comparisons and clinical interpretation [32,33]. On the imaging side, radiomic feature values are highly sensitive to acquisition parameters (scanner vendor, field strength, sequence timing), preprocessing choices, and segmentation methods. Although initiatives such as the Image Biomarker Standardization Initiative have improved feature reproducibility, full harmonization across centers remains incomplete [19,28,59]. Deep-learning models add further variability related to architecture, training strategies, and hardware.

Biological challenges. Not all gliomas release sufficient ctDNA into CSF; lower-grade and certain molecular subtypes may yield false-negative results [13,45]. Spatial heterogeneity means that a single CSF sample reflects an averaged or selectively shed genomic profile that may not capture the full spectrum of clones present in the tumor mass. Radiogenomic signatures, while spatially comprehensive, are indirect phenotypic proxies and can be confounded by peritumoral edema, treatment-related changes, or co-occurring mutations that dilute mutation-specific imaging phenotypes [11,20]. Consequently, perfect concordance between imaging-predicted and CSF-detected alterations cannot be assumed.

Analytical challenges. The majority of radiogenomic and CSF-NGS studies remain retrospective, single-institution, and modest in sample size. Prospective, multi-center validation with predefined endpoints and independent external test sets is still scarce [27,28,43]. Robust multimodal AI models that fuse radiomic features with CSF-NGS data require large, well-annotated paired datasets that are currently limited. Ground-truth correlation with tissue NGS is essential yet imperfect because of sampling bias in the tissue reference itself. Overfitting, lack of calibration, and insufficient reporting of uncertainty further hinder clinical trust in AI outputs [56].

Practical challenges. Access to high-quality multiparametric MRI and specialized CSF-NGS assays is unevenly distributed, particularly outside tertiary centers. Turnaround times for comprehensive CSF sequencing (often days to more than a week) may limit utility in urgent decision-making contexts. Reimbursement pathways for both advanced radiomic analysis and CSF genomic profiling are still evolving and vary by healthcare system; without clear coverage policies, adoption will remain restricted [58]. Integration into existing clinical workflows also requires coordination among neurosurgery, neuroradiology, molecular pathology, and neuro-oncology teams.

Ethical and data-privacy considerations. Multimodal datasets that combine high-resolution imaging, genomic sequences, and longitudinal clinical information raise heightened privacy risks. Even de-identified data can potentially be re-identified when rich imaging and genomic features are linked. Compliance with regulations such as GDPR or HIPAA, secure data-sharing frameworks (including federated learning approaches), transparent consent processes, and equitable access to these technologies are essential. Algorithmic bias—arising from under-representation of certain demographic or molecular subgroups in training data—must be actively monitored to avoid exacerbating disparities in care [58,59].

Collectively, these challenges underscore the need for coordinated standardization efforts: consensus pre-analytical and analytical protocols for CSF-NGS, harmonized MRI acquisition and radiomic pipelines, prospective multi-institutional validation studies, transparent AI reporting standards, and clear regulatory and reimbursement pathways. Addressing these issues will be critical to realizing the clinical potential of integrated radiogenomic and CSF liquid-biopsy approaches.

## 7. Future Directions

The convergent evolution of radiogenomics and CSF-based liquid biopsy sequencing points toward a future in which non- and minimally invasive multimodal profiling becomes central to precision neuro-oncology. Several interrelated avenues will determine how rapidly and effectively this vision is realized.

Prospective multimodal trials are the most immediate priority. Current evidence is largely derived from retrospective or parallel (rather than fully integrated) cohorts. Well-designed prospective studies that systematically collect paired multiparametric MRI, CSF, and tissue at defined clinical landmarks—diagnosis, post-resection, during adjuvant therapy, and at suspected progression—are required to establish the incremental value of combined radiogenomic–CSF-NGS strategies for diagnosis, response assessment, and survival prediction. Such trials should incorporate standardized imaging protocols, harmonized CSF pre-analytical workflows, and predefined clinical endpoints, including differentiation of true progression from pseudoprogression and guidance of adaptive treatment decisions.

Deeper molecular ground truth will come from the incorporation of single-cell, spatial, and long-read sequencing technologies. Single-cell and spatial transcriptomics/multi-omics already reveal the cellular states, niches, and intercellular interactions that drive glioblastoma heterogeneity and treatment resistance. Linking these high-resolution maps to radiomic phenotypes and to the genomic signals detectable in CSF will refine the biological interpretation of imaging and liquid-biopsy biomarkers and may identify new composite signatures of aggressive or therapy-resistant compartments. Long-read (third-generation) sequencing of CSF or tissue can better resolve complex structural variants, extrachromosomal DNA, full-length fusions, and phased haplotypes, providing richer reference data against which short-read CSF-NGS and radiogenomic models can be calibrated.

Real-time adaptive treatment guided by serial radiogenomic and CSF monitoring represents a longer-term but transformative goal. Longitudinal imaging and CSF sampling could enable dynamic risk stratification, early detection of emerging resistance clones (for example, mismatch-repair deficiency after alkylator therapy), and timely therapeutic switches or trial enrollment. Closed-loop systems in which automated radiomic pipelines and rapid CSF-NGS results feed into clinical decision-support tools could ultimately support truly adaptive neuro-oncology protocols.

Multi-omic liquid biopsy—integrating genomics with epigenomics (methylation, fragmentomics), transcriptomics, proteomics, and extracellular-vesicle cargo—will further enrich the CSF signal. When these layered molecular data are fused with multiparametric imaging phenotypes through advanced multimodal artificial-intelligence architectures, the resulting models are expected to achieve higher sensitivity, specificity, and biological interpretability than single-modality approaches. Federated-learning frameworks may allow multi-institutional model training while preserving patient privacy and data sovereignty.

Artificial intelligence will be indispensable for automated clinical decision support. Beyond prediction of individual biomarkers, next-generation systems should generate calibrated, uncertainty-aware integrated scores that assist multidisciplinary teams in diagnosis, monitoring, and therapy selection. Explainability, robustness to domain shifts (scanner, population, treatment era), and seamless embedding into electronic health records and radiology/pathology workflows will be essential for clinical trust and adoption.

The ultimate path toward routine clinical adoption is the maturation of a “virtual + liquid” biopsy paradigm. In this framework, radiogenomics supplies a spatially comprehensive, non-invasive phenotypic overview, while CSF sequencing supplies confirmatory and longitudinal genomic (and multi-omic) detail. Together they can reduce dependence on repeated invasive tissue sampling, expand molecular characterization to patients currently underserved by surgery, and enable more precise, dynamic management of diffuse gliomas. Realization of this paradigm will require sustained interdisciplinary collaboration, investment in infrastructure and standardization, prospective evidence generation, and alignment of regulatory and reimbursement pathways. With these elements in place, the integration of radiogenomics and CSF-based liquid biopsy sequencing has the potential to become a cornerstone of precision neuro-oncology.

**Figure 2:**
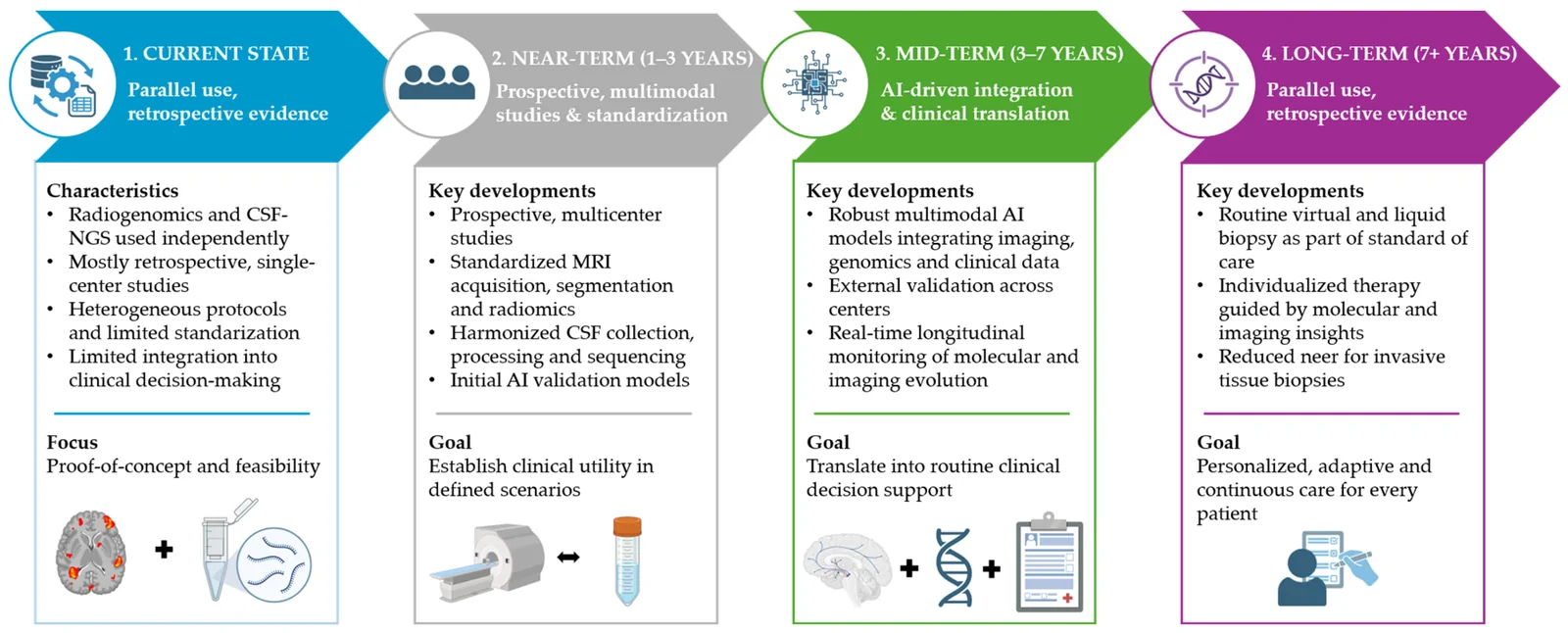
Roadmap toward Clinical Implementation of the Integrated Virtual and Liquid Biopsy Approach. Proposed roadmap for translating integrated radiogenomics and CSF-based next-generation sequencing (CSF-NGS) from retrospective research to routine precision neuro-oncology. Key milestones, enabling technologies, and remaining barriers required for successful clinical implementation are highlighted across successive stages of development. Created in BioRender. Kubiak, K. (2026) https://BioRender.com/l4cqfg8, accessed on 5 August 2026.

Although substantial progress has been achieved, widespread clinical implementation will require harmonized imaging and sequencing protocols, robust multimodal AI models, prospective multicenter validation, and regulatory approval. A proposed roadmap toward routine implementation of the integrated virtual + liquid biopsy approach is presented in Figure 2.

## 8. Conclusions

Radiogenomics and CSF-based liquid biopsy sequencing address complementary limitations of conventional tissue-based molecular profiling in glioblastoma and adult diffuse gliomas. Radiogenomics provides a spatially comprehensive, non-invasive phenotypic characterization of the entire tumor volume, enabling prediction of key NGS-defined alterations and molecular subtypes from multiparametric MRI. CSF-based liquid biopsy sequencing supplies high-fidelity genomic (and increasingly multi-omic) information with superior sensitivity and tissue concordance compared with plasma, supporting diagnosis when tissue is unavailable, monitoring of clonal evolution, detection of therapy resistance, and assessment of residual disease. When integrated, these modalities combine spatial context with temporal genomic resolution, offering a more complete and dynamic view of tumor biology than either approach alone.

Realizing the full clinical potential of this multimodal strategy will require sustained, collaborative efforts across disciplines. Neuro-oncologists, neurosurgeons, neuroradiologists, molecular pathologists, computational scientists, and industry partners must work together to standardize imaging and CSF protocols, generate prospective multi-institutional evidence, develop robust and interpretable artificial-intelligence models, and align regulatory and reimbursement pathways. Only through such coordinated action can technical advances be translated into reliable tools that improve decision-making at the bedside.

Looking ahead, the convergence of radiogenomics and CSF sequencing points toward a future in which “virtual + liquid” biopsy paradigms reduce reliance on repeated invasive procedures, expand molecular characterization to patients currently underserved by surgery, and enable more precise, adaptive management of these aggressive tumors. With continued methodological refinement, rigorous validation, and interdisciplinary commitment, this integrated approach holds substantial promise for advancing precision neuro-oncology and ultimately improving outcomes for patients with glioblastoma and related diffuse gliomas. Importantly, the proposed integrated framework should currently be regarded as a promising investigational strategy rather than an established clinical standard, as most radiogenomic models remain insufficiently externally validated and prospective multicenter evidence demonstrating clinical utility is still limited.

## Figures and Tables

**Table 1:** Performance of radiogenomic models for major NGS-defined biomarkers in gliomas.

Biomarker	Clinical Significance	Radiogenomic AUC (Internal/External)	Key MRI Sequences & Radiomic Features	AI/ML Approaches	Main Limitations
*IDH1/2* mutation [30,31,32,33]	WHO classification, prognosis, treatment stratification	0.82–0.95/ 0.75–0.90	T2/FLAIR, DWI, DSC perfusion, texture, habitat analysis	Random Forest, SVM, CNN, Vision Transformers	Scanner variability
*MGMT* promoter methylation [34,35,36,37]	Predicts temozolomide response	0.70–0.88/ 0.65–0.82	Contrast enhancement, ADC, perfusion, texture	Elastic Net, XGBoost, CNN	Moderate reproducibility
*EGFR* amplification/*EGFRvIII* [38,39,40]	Targeted therapy, aggressive phenotype	0.72–0.90/ 0.68–0.82	Perfusion, necrotic volume, enhancement heterogeneity	CNN, Random Forest	Biological heterogeneity
*TERT* promoter mutation [41,42]	Prognosis, molecular classification	0.70–0.87/ 0.65–0.80	Texture, shape, tumor location	ML, deep learning	Limited cohorts
*CDKN2A/B* deletion [12]	WHO CNS5 grading, prognosis	0.68–0.82/ limited external validation	Tumor border irregularity, FLAIR infiltration	Random Forest, Gradient Boosting	Sparse literature
Transcriptomic/Molecular subtypes [30,32,43]	Precision therapy, biological stratification	0.70–0.90	Multiparametric MRI, habitat imaging	Deep CNN, multimodal AI	Lack of standardized labels

Performance estimates vary substantially owing to differences in MRI acquisition protocols, segmentation strategies, radiomic feature harmonization, feature selection methods, AI architectures, cohort size, molecular testing platforms, and the availability of external validation. Direct comparison between studies should therefore be interpreted with caution.

**Table 2:** CSF-ctDNA Detection Rates, Tissue Concordance, and Common Genomic Alterations in Adult Diffuse Gliomas.

Study	Cohort Characteristics	CSF Detection Rate	Plasma Detection Rate	Tissue–CSF Concordance	Most Frequent Alterations Detected
Systematic review & meta-analysis (2025) [14]	12 studies; 388 adult WHO grade II–IV gliomas	82%	16%	90%	*IDH1/2*, *TP53*, *EGFR*, *PTEN*, *TERT* promoter, *ATRX*, *PIK3CA*, *CDKN2A/B*, CNVs
Multicenter prospective study (2025) [13]	37 patients (32 evaluable); newly diagnosed and recurrent diffuse gliomas; paired CSF, plasma and tumor sequencing	59%	14%	84%	*EGFR* (42%), *PTEN* (37%), *TP53* (32%), *IDH1* (26%), *PIK3CA* (21%)
Integrated cfDNA/cfRNA prospective study (2025) [47]	71 CSF samples from adult gliomas; targeted cfDNA/cfRNA sequencing	77% (cfDNA)	NR	89%	*IDH1/2*, *TP53*, *EGFR*, *PTEN*, *ATRX*, *TERT* promoter, *BRAF*, *CDKN2A/B*
Prospective NGS concordance study (2023) [51]	27 paired lumbar CSF and tumor samples; WHO 2021 integrated diagnosis	89%	NR	91.6%	*IDH1/2*, *TERT* promoter, *EGFR*, *TP53*, chromosome alterations
Systematic review & meta-analysis (2022) [52]	Adult glioma ctDNA studies (CSF and plasma)	Higher performance in CSF than plasma	Lower sensitivity in plasma	—	Predominantly *IDH1/2*, *TERT* promoter, *EGFR*, *TP53*
Diagnostic meta-analysis (2020) [53]	11 studies; 522 glioma patients	Variable (higher in CSF than plasma)	Variable	Compared with tumor tissue	Lack of standardized labels

Detection rates vary according to tumor grade, tumor proximity to CSF spaces, timing of sampling (preoperative vs. recurrence), sequencing platform (targeted panels, WES, ddPCR), pre-analytical processing, and the definition of ctDNA positivity. Reported values should therefore be interpreted as representative rather than directly comparable across studies. CNV: copy number variation; CSF: cerebrospinal fluid; ctDNA: circulating tumor DNA; NGS: next-generation sequencing; NR: not reported.

**Table 3:** Complementary strengths and limitations of radiogenomics versus CSF-based liquid biopsy sequencing in adult diffuse gliomas.

Feature	Virtual Biopsy	Liquid Biopsy	Integrated Approach
Primary information	Imaging-derived spatial phenotype and inferred molecular characteristics	Direct genomic and epigenomic characterization of tumor-derived nucleic acids	Combines spatial and molecular information
Spatial coverage	Excellent—whole tumor, infiltrative margins, edema, multifocal disease	Limited anatomical localization; reflects DNA shed into CSF	MRI localizes abnormalities while CSF confirms molecular alterations
Temporal resolution	High; repeat MRI during routine follow-up	High; serial CSF sampling enables longitudinal molecular monitoring	Dynamic monitoring of imaging and molecular evolution
Invasiveness	Completely non-invasive	Minimally invasive (lumbar puncture or ventricular sampling)	Reduced need for repeat surgical biopsy
Sensitivity for molecular alterations	Indirect prediction based on imaging phenotype	Direct detection of SNVs, indels, CNVs, gene fusions, methylation, cfRNA	Imaging predicts; NGS confirms
Intratumoral heterogeneity	Excellent spatial characterization using radiomic habitats and advanced MRI	Detects genomic heterogeneity but lacks spatial localization	Spatial and molecular heterogeneity assessed simultaneously
Monitoring treatment response	Detects anatomical and physiological changes	Detects ctDNA dynamics and emerging resistance	Earlier recognition of treatment failure
Pseudoprogression vs. true progression	Moderate accuracy despite advanced MRI	Molecular evidence may support discrimination	Combined approach expected to improve diagnostic confidence
Minimal residual disease (MRD)	Limited sensitivity	High potential via ultrasensitive ctDNA detection	Combined imaging and molecular surveillance
Clinical readiness	Moderate; several externally validated models but limited standardization	Moderate; promising prospective evidence but mainly research use	Emerging precision neuro-oncology workflow
Major technical challenges	MRI harmonization, segmentation variability, feature reproducibility, AI generalizability	Low ctDNA abundance, pre-analytical variability, assay standardization	Data harmonization, multimodal AI integration, prospective validation
Future role	Virtual biopsy	Molecular surveillance	Continuous multimodal disease characterization

**Table 4:** Priority Clinical Scenarios for Integrated Radiogenomic + CSF-NGS Applications in Adult Diffuse Gliomas.

Clinical Scenario	Primary Contribution of Radiogenomics (MRI + AI)	Primary Contribution of CSF-NGS	Expected Added Value of Integration	Evidence Level/ Clinical Readiness
Initial diagnosis and molecular classification	Non-invasive prediction of key molecular features (*IDH1/2* status, *MGMT* methylation, *EGFR* alterations, molecular subtype), tumor localization, and characterization of infiltrative phenotype	Detection of tumor-derived alterations including SNVs, indels, CNVs, fusions, and methylation signatures when tissue is limited or unavailable	Enables a “virtual + liquid biopsy” approach to support integrated WHO CNS5 classification and reduce dependence on repeat invasive sampling	Moderate evidence; emerging clinical implementation
Treatment planning and patient stratification	Identifies spatial heterogeneity, tumor habitats, enhancing and non-enhancing regions, and potential regions of aggressive biology for targeted sampling or treatment planning	Provides molecular profiling of actionable alterations and pathway activation relevant for targeted therapies and clinical trial eligibility	Improves selection of patients for molecularly guided therapy and allows imaging-based spatial context to complement genomic information	Emerging evidence; investigational clinical use
Monitoring treatment response and distinguishing pseudoprogression from true progression	Serial MRI detects anatomical and physiological changes using conventional MRI, perfusion, diffusion, spectroscopy, and radiomic biomarkers	Serial CSF-NGS detects changes in ctDNA levels, emerging resistance alterations, clonal evolution, and molecular progression	Combining imaging evolution with molecular dynamics may improve early discrimination between treatment effect and tumor progression	Promising but requires prospective validation
Minimal residual disease (MRD) detection and recurrence surveillance	Detects radiographic recurrence, subtle changes in tumor phenotype, and spatial patterns of relapse	Provides molecular evidence of residual disease, recurrence-associated alterations, and early clonal expansion before overt imaging progression	Enables earlier detection of recurrence and may allow pre-emptive therapeutic intervention before radiographic progression	Early clinical evidence; investigational
Tracking clonal evolution and therapy resistance	Maps spatial evolution of tumor regions, including changes in tumor habitat and infiltrative behavior	Directly identifies newly emerging mutations, hypermutation signatures, copy-number alterations, and resistance mechanisms	Creates a longitudinal model of tumor adaptation integrating where evolution occurs with which molecular events drive it	Early evidence; research setting
Clinical trial stratification and adaptive trial design	Enables imaging-based enrichment of biologically defined populations and assessment of spatial treatment response	Provides molecular eligibility criteria, pharmacodynamic biomarkers, and real-time monitoring of treatment effects	Facilitates biomarker-driven trials, adaptive treatment strategies, and patient-specific response assessment	Conceptual framework; limited prospective evidence
Radiation and local therapy planning	Identifies biologically aggressive regions, hypoxic areas, and infiltrative margins for dose adaptation or targeted interventions	Provides molecular features associated with radioresistance and tumor evolution	Integration may enable biologically informed radiation planning and adaptive treatment approaches	Preclinical/early translational evidence

## Data Availability

No new data were created or analyzed in this study. Data sharing is not applicable to this article.

## Abbreviations

The following abbreviations are used in this manuscript:

ADC	Apparent diffusion coefficient
AI	Artificial intelligence
AUC	Area under the curve
BRAF	B-Raf proto-oncogene
CDKN2A/B	Cyclin-dependent kinase inhibitor 2A/B
cfDNA/cfRNA	Cell-free DNA/cell-free RNA
CNN	Convolutional neural network
CNS	Central nervous system
CNV	Copy-number variation
CSF	Cerebrospinal fluid
ctDNA/ctRNA	Circulating tumor DNA/circulating tumor RNA
DCE	Dynamic contrast-enhanced
DSC	Dynamic susceptibility contrast
DTI	Diffusion tensor imaging
DWI	Diffusion-weighted imaging
EGFR/EGFRvIII	Epidermal growth factor receptor/EGFR variant III
FLAIR	Fluid-attenuated inversion recovery
GBM	Glioblastoma
GDPR	General Data Protection Regulation
HIPAA	Health Insurance Portability and Accountability Act
IDH	Isocitrate dehydrogenase
MGMT	O^6^-methylguanine-DNA methyltransferase
ML	Machine learning
MMR	Mismatch repair
MRD	Minimal residual disease
MRI	Magnetic resonance imaging
MRS	Magnetic resonance spectroscopy
NCCN	National Comprehensive Cancer Network
NGS	Next-generation sequencing
NR	Not reported
PTEN	Phosphatase and tensin homolog
SNV	Single-nucleotide variant
SVM	Support vector machine
TERT	Telomerase reverse transcriptase
TP53	Tumor protein p53
VAF	Variant allele frequency
WES	Whole-exome sequencing
WHO CNS2021	WHO Classification of Central Nervous System Tumors (5th edition)
XGBoost	Extreme Gradient Boosting

## References

[1] Wen P.Y., Weller M., Lee E.Q., Touat M., Khasraw M., Rahman R., Platten M., Lim M., Winkler F., Horbinski C. (2025). Glioblastoma in Adults: A Society for Neuro-Oncology (SNO) and European Society of Neuro-Oncology (EANO) Consensus Review on Current Management and Future Directions. Neuro-Oncology.

[2] Louis D.N., Perry A., Wesseling P., Brat D.J., Cree I.A., Figarella-Branger D., Hawkins C., Ng H.K., Pfister S.M., Reifenberger G.W. (2021). The 2021 WHO Classification of Tumors of the Central Nervous System: A Summary. Neuro-Oncology.

[3] Neftel C., Laffy J., Filbin M.G., Hara T., Shore M.E., Rahme G.J., Richman A.R., Silverbush D., Shaw M.L., Hebert C.M. (2019). An Integrative Model of Cellular States, Plasticity, and Genetics for Glioblastoma. Cell.

[4] Nomura M., Spitzer A., Johnson K.C., Garofano L., Nehar-Belaid D., Galili Darnell N., Greenwald A.C., Bussema L., Oh Y.T., Varn F.S. (2025). The Multilayered Transcriptional Architecture of Glioblastoma Ecosystems. Nat. Genet..

[5] Mathur R., Wang Q., Schupp P.G., Nikolic A., Hilz S., Hong C., Grishanina N.R., Kwok D., Stevers N.O., Jin Q. (2024). Glioblastoma Evolution and Heterogeneity from a 3D Whole-Tumor Perspective. Cell.

[6] Capper D., Jones D.T.W., Sill M., Hovestadt V., Schrimpf D., Sturm D., Koelsche C., Sahm F., Chavez L., Reuss D. (2018). DNA Methylation-Based Classification of Central Nervous System Tumours. Nature.

[7] Ontario Health (Quality) (2025). DNA Methylation-Based Classification for Central Nervous System Tumours: A Health Technology Assessment. Ont. Health Technol. Assess. Ser..

[8] Karschnia P., Smits M., Reifenberger G., Le Rhun E., Ellingson B.M., Galldiks N., Kim M.M., Huse J.T., Schnell O., Harter P.N. (2023). A Framework for Standardised Tissue Sampling and Processing during Resection of Diffuse Intracranial Glioma: Joint Recommendations from Four RANO Groups. Lancet Oncol..

[9] Sottoriva A., Spiteri I., Piccirillo S.G.M., Touloumis A., Collins V.P., Marioni J.C., Curtis C., Watts C., Tavaré S. (2013). Intratumor Heterogeneity in Human Glioblastoma Reflects Cancer Evolutionary Dynamics. Proc. Natl. Acad. Sci. USA.

[10] Singh K., Hotchkiss K.M., Parney I.F., De Groot J., Sahebjam S., Sanai N., Platten M., Galanis E., Lim M., Wen P.Y. (2023). Correcting the Drug Development Paradigm for Glioblastoma Requires Serial Tissue Sampling. Nat. Med..

[11] Fathi Kazerooni A., Akbari H., Hu X., Bommineni V., Grigoriadis D., Toorens E., Sako C., Mamourian E., Ballinger D., Sussman R. (2025). The Radiogenomic and Spatiogenomic Landscapes of Glioblastoma and Their Relationship to Oncogenic Drivers. Commun. Med..

[12] Yan J., Zhang B., Zhang S., Cheng J., Liu X., Wang W., Dong Y., Zhang L., Mo X., Chen Q. (2021). Quantitative MRI-based radiomics for noninvasively predicting molecular subtypes and survival in glioma patients. npj Precis. Oncol..

[13] Cabezas-Camarero S., Pérez-Alfayate R., García-Barberán V., Gandía-González M.L., García-Feijóo P., López-Cade I., Lorca V., Casado-Fariñas I., Cerón M.A., Paz-Cabezas M. (2025). CtDNA Detection in Cerebrospinal Fluid and Plasma and Mutational Concordance with the Primary Tumor in a Multicenter Prospective Study of Patients with Glioma. Ann. Oncol..

[14] Pérez-Alfayate R., Paz-Cabezas M., Pérez-Segura P., Sanchez del Hoyo R., Cabezas-Camarero S. (2025). Cerebrospinal Fluid CtDNA as a Diagnostic and Prognostic Tool in Gliomas: A Systematic Review and Meta-Analysis. Front. Oncol..

[15] Miller A.M., Szalontay L., Bouvier N., Hill K., Ahmad H., Rafailov J., Lee A.J., Rodriguez-Sanchez M.I., Yildirim O., Patel A. (2022). Next-generation sequencing of cerebrospinal fluid for clinical molecular diagnostics in pediatric, adolescent and young adult brain tumor patients. Neuro-Oncology.

[16] Miller A.M., Shah R.H., Pentsova E.I., Pourmaleki M., Briggs S., Distefano N., Zheng Y., Skakodub A., Mehta S.A., Campos C. (2019). Tracking Tumour Evolution in Glioma through Liquid Biopsies of Cerebrospinal Fluid. Nature.

[17] Awais M., Rehman A., Bukhari S.S. (2025). Advances in Liquid Biopsy and Virtual Biopsy for Care of Patients with Glioma: A Narrative Review. Expert Rev. Anticancer Ther..

[18] Zwanenburg A., Vallières M., Abdalah M.A., Aerts H.J.W.L., Andrearczyk V., Apte A., Ashrafinia S., Bakas S., Beukinga R.J., Boellaard R. (2020). The Image Biomarker Standardization Initiative: Standardized Quantitative Radiomics for High-Throughput Image-Based Phenotyping. Radiology.

[19] Gillies R.J., Kinahan P.E., Hricak H. (2016). Radiomics: Images Are More than Pictures, They Are Data. Radiology.

[20] Guo Y., Li T., Gong B., Hu Y., Wang S., Yang L., Zheng C. (2025). From Images to Genes: Radiogenomics Based on Artificial Intelligence to Achieve Non-Invasive Precision Medicine in Cancer Patients. Adv. Sci..

[21] Lambin P., Rios-Velazquez E., Leijenaar R., Carvalho S., Van Stiphout R.G.P.M., Granton P., Zegers C.M.L., Gillies R., Boellard R., Dekker A. (2012). Radiomics: Extracting More Information from Medical Images Using Advanced Feature Analysis. Eur. J. Cancer.

[22] Calabrese E., Rudie J.D., Rauschecker A.M., Villanueva-Meyer J.E., Clarke J.L., Solomon D.A., Cha S. (2022). Combining Radiomics and Deep Convolutional Neural Network Features from Preoperative MRI for Predicting Clinically Relevant Genetic Biomarkers in Glioblastoma. Neuro-Oncol. Adv..

[23] Nakase T., Henderson G.A., Barba T., Bareja R., Guerra G., Zhao Q., Francis S.S., Gevaert O., Kachuri L. (2025). Integration of MRI Radiomics and Germline Genetics to Predict the IDH Mutation Status of Gliomas. npj Precis. Oncol..

[24] Sohn B., Park K., Ahn S.S., Park Y.W., Choi S.H., Kang S.G., Kim S.H., Chang J.H., Lee S.K. (2023). Dynamic Contrast-Enhanced MRI Radiomics Model Predicts Epidermal Growth Factor Receptor Amplification in Glioblastoma, IDH-Wildtype. J. Neuro-Oncol..

[25] Chen L., Chen R., Li T., Tang C., Li Y., Zeng Z. (2023). Multi-Parameter MRI Based Radiomics Nomogram for Predicting Telomerase Reverse Transcriptase Promoter Mutation and Prognosis in Glioblastoma. Front. Neurol..

[26] Astaraki M., Lazzeroni M., Toma-Dasu I. (2026). A Comprehensive Evaluation of MRI-Based Radiogenomics and Prognosis Prediction in Glioma. Front. Oncol..

[27] Kocak B., Banaz T., Soyleyici M., Kose F., Keles A., Mese I. (2026). Externally Validated yet Undertrained: Sample Size Deficits in Machine Learning-Based Radiomics. Eur. Radiol..

[28] Santinha J., Matos C., Figueiredo M., Papanikolaou N. (2021). Improving Performance and Generalizability in Radiogenomics: A Pilot Study for Prediction of IDH1/2 Mutation Status in Gliomas with Multicentric Data. J. Med. Imaging.

[29] Ismail M., Craig S., Ahmed R., de Blank P., Tiwari P. (2023). Opportunities and Advances in Radiomics and Radiogenomics for Pediatric Medulloblastoma Tumors. Diagnostics.

[30] Verhaak R.G.W., Hoadley K.A., Purdom E., Wang V., Qi Y., Wilkerson M.D., Miller C.R., Ding L., Golub T., Mesirov J.P. (2010). Integrated Genomic Analysis Identifies Clinically Relevant Subtypes of Glioblastoma Characterized by Abnormalities in PDGFRA, IDH1, EGFR, and NF1. Cancer Cell.

[31] Choi Y.S., Ahn S.S., Chang J.H., Kang S.G., Kim E.H., Kim S.H., Jain R., Lee S.K. (2020). Machine Learning and Radiomic Phenotyping of Lower Grade Gliomas: Improving Survival Prediction. Eur. Radiol..

[32] Bakas S., Akbari H., Sotiras A., Bilello M., Rozycki M., Kirby J.S., Freymann J.B., Farahani K., Davatzikos C. (2017). Advancing The Cancer Genome Atlas Glioma MRI Collections with Expert Segmentation Labels and Radiomic Features. Sci. Data.

[33] Kickingereder P., Burth S., Wick A., Götz M., Eidel O., Schlemmer H.P., Maier-Hein K.H., Wick W., Bendszus M., Radbruch A. (2016). Radiomic Profiling of Glioblastoma: Identifying an Imaging Predictor of Patient Survival with Improved Performance over Established Clinical and Radiologic Risk Models. Radiology.

[34] Abdelaziz N.M., Dawood E.A.A., Tantawy A.A. (2025). Integrating Deep Learning and Radiogenomics: A Novel Approach to Glioblastoma Segmentation and MGMT Methylation Prediction. J. Imaging.

[35] Leone A., Di Napoli V., Fochi N.P., Di Perna G., Spetzger U., Filimonova E., Angileri F., Carbone F., Colamaria A. (2025). Virtual Biopsy for the Prediction of MGMT Promoter Methylation in Gliomas: A Comprehensive Review of Radiomics and Deep Learning Approaches Applied to MRI. Diagnostics.

[36] Yu X., Zhou J., Wu Y., Bai Y., Meng N., Wu Q., Jin S., Liu H., Li P., Wang M. (2024). Assessment of MGMT Promoter Methylation Status in Glioblastoma Using Deep Learning Features from Multi-Sequence MRI of Intratumoral and Peritumoral Regions. Cancer Imaging.

[37] Faghani S., Khosravi B., Moassefi M., Conte G.M., Erickson B.J. (2023). A Comparison of Three Different Deep Learning-Based Models to Predict the MGMT Promoter Methylation Status in Glioblastoma Using Brain MRI. J. Digit. Imaging.

[38] Hu L.S., Wang L., Hawkins-Daarud A., Eschbacher J.M., Singleton K.W., Jackson P.R., Clark-Swanson K., Sereduk C.P., Peng S., Wang P. (2021). Uncertainty Quantification in the Radiogenomics Modeling of EGFR Amplification in Glioblastoma. Sci. Rep..

[39] Beig N., Patel J., Prasanna P., Hill V., Gupta A., Correa R., Bera K., Singh S., Partovi S., Varadan V. (2018). Radiogenomic Analysis of Hypoxia Pathway Is Predictive of Overall Survival in Glioblastoma. Sci. Rep..

[40] Akbari H., Macyszyn L., Da X., Bilello M., Wolf R.L., Martinez-Lage M., Biros G., Alonso-Basanta M., O’Rourke D.M., Davatzikos C. (2016). Imaging Surrogates of Infiltration Obtained via Multiparametric Imaging Pattern Analysis Predict Subsequent Location of Recurrence of Glioblastoma. Neurosurgery.

[41] Tang C., Chen L., Xu Y., Huang L., Zeng Z. (2024). Prediction of TERT Mutation Status in Gliomas Using Conventional MRI Radiogenomic Features. Front. Neurol..

[42] Ivanidze J., Lum M., Pisapia D., Magge R., Ramakrishna R., Kovanlikaya I., Fine H.A., Chiang G.C. (2019). MRI Features Associated with TERT Promoter Mutation Status in Glioblastoma. J. Neuroimaging.

[43] Guarnera A., Ius T., Romano A., Bagatto D., Denaro L., Aiudi D., Iacoangeli M., Palmieri M., Frati A., Santoro A. (2025). Advanced MRI, Radiomics and Radiogenomics in Unravelling Incidental Glioma Grading and Genetic Status: Where Are We?. Medicina.

[44] Bagley S.J., Beaubier N., Balaj L., Bettegowda C., Carpenter E., Carter B.S., Corner A., Dittamore R., Grossman R.L., Hickman R.A. (2025). Minimum Technical Preanalytical, Patient, and Clinical Context Data Elements for Cerebrospinal Fluid Liquid Biopsy: Recommendations for Public Database Submissions. JCO Precis. Oncol..

[45] Mouliere F., Chandrananda D., Piskorz A.M., Moore E.K., Morris J., Ahlborn L.B., Mair R., Goranova T., Marass F., Heider K. (2018). Enhanced Detection of Circulating Tumor DNA by Fragment Size Analysis. Sci. Transl. Med..

[46] Piccioni D.E., Achrol A.S., Kiedrowski L.A., Banks K.C., Boucher N., Barkhoudarian G., Kelly D.F., Juarez T., Lanman R.B., Raymond V.M. (2019). Analysis of Cell-Free Circulating Tumor DNA in 419 Patients with Glioblastoma and Other Primary Brain Tumors. CNS Oncol..

[47] Sun M., Jiang S., Xu H., Zeng Y., Liu B., Wang Y., Li Z., Guan Y., Chen J., Zhu R. (2026). Integrated Detection of Cerebrospinal Fluid CfDNA/CfRNA and Molecular Concordance with Glioma Characteristics. Neuro-Oncol. Adv..

[48] Touat M., Li Y.Y., Boynton A.N., Spurr L.F., Iorgulescu J.B., Bohrson C.L., Cortes-Ciriano I., Birzu C., Geduldig J.E., Pelton K. (2020). Mechanisms and Therapeutic Implications of Hypermutation in Gliomas. Nature.

[49] Abuhassan Q., Ahmed H.H., Kareem R.A., Menon S.V., Nayak P.P., Janney J.B., Arora V., Sinha A., Jaber S.A., Sameer H.N. (2026). The Clinical Utility of Cell-Free DNA in Brain Tumor Management: A Comprehensive Review. J. Mol. Neurosci..

[50] Wang Z., Jiang W., Wang Y., Guo Y., Cong Z., Du F., Song B. (2015). MGMT promoter methylation in serum and cerebrospinal fluid as a tumor-specific biomarker of glioma. Biomed. Rep..

[51] Wang Q., Liang Q., Wei W., Niu W., Liang C., Wang X., Wang X., Pan H. (2023). Concordance Analysis of Cerebrospinal Fluid with the Tumor Tissue for Integrated Diagnosis in Gliomas Based on Next-Generation Sequencing. Pathol. Oncol. Res..

[52] McMahon J.T., Studer M., Ulrich B., Revuelta Barbero J.M., Pradilla I., Palacios-Ariza M.A., Pradilla G. (2022). Circulating Tumor DNA in Adults with Glioma: A Systematic Review and Meta-Analysis of Biomarker Performance. Neurosurgery.

[53] Kang Y., Lin X., Kang D. (2020). Diagnostic Value of Circulating Tumor DNA in Molecular Characterization of Glioma A Meta-Analysis. Medicine.

[54] Leary O.P., Ma Z., Hwang H., Pizzagalli M.D., Zhong Z., Tran L., Voong V., Choi A., Arditi J., Zhu M. (2026). miRNA Liquid Biopsy Combined with MRI Radiomics for Improved Outcome Prediction in Glioblastoma: Integrated Machine Learning Analysis of Longitudinal Data from 73 Patients. Neuro-Oncol. Adv..

[55] Hu P., Xu L., Qi Y., Yan T., Ye L., Wen S., Yuan D., Zhu X., Deng S., Liu X. (2023). Combination of Multi-Modal MRI Radiomics and Liquid Biopsy Technique for Preoperatively Non-Invasive Diagnosis of Glioma Based on Deep Learning: Protocol for a Double-Center, Ambispective, Diagnostical Observational Study. Front. Mol. Neurosci..

[56] Bakas S., Vollmuth P., Galldiks N., Booth T.C., Aerts H.J.W.L., Bi W.L., Wiestler B., Tiwari P., Pati S., Baid U. (2024). Artificial Intelligence for Response Assessment in Neuro Oncology (AI-RANO), Part 2: Recommendations for Standardisation, Validation, and Good Clinical Practice. Lancet Oncol..

[57] Gajjar A., Mahajan A., Bale T., Bowers D.C., Canan L., Chi S., Cluster A., Cohen K., Cole B., Coven S. (2025). Pediatric Central Nervous System Cancers, Version 2.2025, NCCN Clinical Practice Guidelines in Oncology. J. Natl. Compr. Cancer Netw..

[58] Martínez R.M., de Marco A., Blanquer I., Martí-Bonmatí L. (2026). Building Trust and Privacy in Cross-Border Health Data Sharing for European Cancer Research. Radiol. Adv..

[59] Khalighi S., Reddy K., Midya A., Pandav K.B., Madabhushi A., Abedalthagafi M. (2024). Artificial Intelligence in Neuro-Oncology: Advances and Challenges in Brain Tumor Diagnosis, Prognosis, and Precision Treatment. npj Precis. Oncol..

